# Aphids May Facilitate the Spread of Sclerotinia Stem Rot in Oilseed Rape by Carrying and Depositing Ascospores

**DOI:** 10.3390/jof10030202

**Published:** 2024-03-06

**Authors:** Zhong-Ping Hao, Lei Sheng, Zeng-Bei Feng, Wei-Xin Fei, Shu-Min Hou

**Affiliations:** Crop Research Institute, Anhui Academy of Agricultural Sciences, Hefei 230031, China; hzp5187@sina.com (Z.-P.H.); 13685514362@163.com (L.S.); zaoanxun@163.com (Z.-B.F.); fei_weixin@163.com (W.-X.F.)

**Keywords:** *Breveroryne brassicae*, *Sclerotinia sclerotiorum*, oilseed rape, feeding behavior, the electric penetration graph, resistance

## Abstract

Aphids and Sclerotinia stem rot in oilseed rape are often studied in isolation, and their relationship is rarely explored. Our field studies have revealed a significant positive correlation between the number of aphids and the incidence of Sclerotinia stem rot. Hence, starting with the colonizing stages of the two pests, *Breveroryne brassicae* was assessed for its potential to acquire, transmit, and inoculate *Sclerotinia sclerotiorum* by being sprayed with an ascospore suspension. Moreover, distinctions in aphid feeding behavior were examined between aphids on inoculated/uninoculated winter and spring oilseed rape plants or aphids, both with and without *S. sclerotiorum* ascospores, using electropenetrography (EPG). The results showed that aphid feeding followed by dropping ascospore suspension significantly increased the incidence of *S. sclerotiorum*. Ascospores were able to adhere to aphids and were carried by aphids to healthy plants, causing disease. The results of the EPG analysis indicated that aphid feeding behavior was significantly altered in all leaf tissue levels following infection with *S. sclerotiorum*. Specifically, aphids initiated their first puncture significantly sooner, began probing mesophyll cells earlier, had a significantly shorter pathway duration, and secreted saliva more frequently but reduced salivation prior to feeding and ingestion compared to aphids feeding on uninfected oilseed rape. Additionally, the feeding behavior of aphids carrying ascospores was markedly different from that of aphids not carrying ascospores, implying that ascospores directly influence aphid feeding behavior but that this influence appeared to be beneficial only for *S. sclerotiorum* infection. Aphids carrying ascospores started to puncture cells more quickly, with a significant increase in the frequency and duration of short probes and cell punctures, shortened pathway durations, and reduced salivation before feeding compared to aphids not carrying ascospores. It is clear that there is an interaction between aphids and *S. sclerotiorum*. The impact of *S. sclerotiorum* on aphid feeding behavior is directional, favoring the spread of the fungus.

## 1. Introduction

Oilseed rape (*Brassica napus* Linnaeus) is a significant oilseed crop worldwide [1]. It can be grown as a winter or spring crop. In China, aphids and Sclerotinia stem rot (SSR) are the primary factors that reduce oilseed rape yield [2]. Aphids consume plant sap, which can result in stunted growth. They can also transmit various viruses, including those that affect the *Brassicaceae* family. Additionally, aphids excrete honeydew, which can lead to fungal growth [3]. The occurrence of the plant disease referred to as SSR is attributable to the necrotrophic pathogen *Sclerotinia sclerotiorum* Lib. (de Bary), which invades the cotyledons and leaves of seedlings, as well as the stems and leaves of mature plants. As a result, characteristic indications manifest in the form of water-soaked lesions, necrotic tissues exhibiting fluffy white mycelium, and the presence of sclerotia within the stems [4,5].

*Sclerotinia sclerotiorum* is dispersed through airborne ascospores or soilborne sclerotia. Carpogenic germination is characterized by the release of thousands of airborne spores (ascospores, size 9–14 μm × 3–6 μm) from fruiting bodies called apothecia [6,7]. The fresh ascospores can survive for 2 to 3 weeks, depending on environmental conditions [8]. *Sclerotinia sclerotiorum* spores can only germinate and invade the host when deposited on a surface with exogenous nutrients, such as senescent petals [9,10]. Although some ascospores germinate into the stomata or wound as diffuse substances, mycelium invasion can only occur through the stratum corneum using enzymes or mechanical force [11,12]. Petal infection is a crucial stage in the cycle of SSR [13]. Additionally, *S. sclerotiorum* can be transmitted through insects, fieldwork, or sap [14]. Research conducted by Dillard and Cobb [15] found that *S. sclerotiorum* does not infect *Brassicaceae* without mechanical damage or insect feeding damage. Moreover, the average infection rate of *S. sclerotiorum* at feeding locations of lepidopteran larvae ranges from 5 to 60%. Although there are rare studies in this area, it appears that phytophagous insects may be able to promote *S. sclerotiorum* infection.

Yamoah [16] suggested that insects could potentially aid in the spread of fungal infection through three primary mechanisms: transmitting pathogen spores, generating sites of injury for fungal infiltration, or establishing a suitable environment for fungal growth and development. Numerous herbivorous and nectar-feeding insects act as vectors for plant pathogens (as cited in [17]). Gao et al. [18] found that *S. sclerotiorum* can be transmitted by insects such as the honeybee *Apis cerana* (Fabricus) (*Hymenoptera: Apidae*) and the cabbage butterfly *Pieris rapae* (L.) (*Lepidoptera: Pieridae*) through experiments involving culturing their bodies and eluent, obtaining sclerotia, and re-culturing and inoculating the obtained sclerotia.

Insects can cause various degrees of physical damage to plant tissues through feeding, egg-laying, and habitat search. Damaged plant tissues are often more susceptible to fungal infection than healthy tissues because wounds provide easy access for fungal invasion [16,19]. *Ceutorhynchidius troglodytes* (Fabricius) (*Coleoptera: Curculionidae*) can facilitate the spread of disease by creating wounds that allow the pathogen *Phomopsis subordinaria* (Desmazières) to enter the plant host *Plantago lanceolata* (L.) (*Plantaginaceae*) [20]. In Europe, *Lobesia botrana* (Denis & Schiffermüller) (European grapevine moth; *Lepidoptera: Tortricidae*) significantly increases the severity of Botrytis bunch rot. Larvae carrying spores of *B. cinerea* (Pers.: Fr) can infect grape berries by tunneling through them to feed, creating wounds, and facilitating colonization by spores on the berry surface [21,22,23].

Klein and Auld [24] noted that insect-induced damage can cause plant cells to rupture, releasing water and nutrients. They also found that *Colletotrichum orbiculare* (Berkeley and Montagne) infection on *Xanthium spinosum* (L.) (*Asteraceae*: *Compositae*) can be more severe in plants that have been damaged by insects. Insects may create a favorable environment for fungal infection. Furthermore, both whitefly larvae and adults ingest plant cell sap and secret excess sugar onto leaves in the form of honeydew. This substance can be utilized by the fungus *Metacapnodium fraserae* (S. Hughes) to form molds on leaves and fruits [25], which reduces the photosynthetic capacity of the leaves and negatively impacts plant growth [26].

Aphids are pests with a piercing-sucking mouthpart. Their feeding behavior results in numerous wounds on the plant and the secretion of a small amount of gelatinous saliva on the plant surface [27]. During the insertion of the stylets into the intercellular spaces of the plant epidermis and the mesophyll tissues, the sheath, as described by Tjallingii [27] and Tjallingii and Hogen-Esch [28], is formed and quickly hardens to protect the stylets from mechanical damage and insulate the plant from chemical attack. When the stylet probes the sieve tube molecules, the aphid secretes a large amount of water-soluble saliva into the molecules and begins to ingest the sap from the sieve tube. The aphid intermittently secretes water-soluble saliva that mixes with the sieve tube sap [27]. One of the main functions of water-soluble saliva is to inhibit the plant’s defense response to feeding [29]. Furthermore, aphids secrete honeydew on plant leaves [30], creating favorable conditions for fungal infection.

In recent years, due to the increasing planting density of farmland rapeseed resulting from improved farming practices, the occurrence of *S. sclerotiorum* has been facilitated to some extent [12]. Although SSR is more likely to occur in cool and wet conditions [11], spore dispersal usually happens in warm and dry conditions with wind [8]. High temperatures, both locally and externally, as well as high humidity, are favorable for aphids and *S. sclerotiorum* [31]. It is hypothesized that aphids play an important role in facilitating and complementing the transmission and infection of *S. sclerotiorum*. Understanding the interactions between aphids and *S. sclerotiorum* is crucial for developing management strategies for both the insect pest and *S. sclerotiorum* of oilseed rape. To investigate the potential associations between aphids and *S. sclerotiorum*, we examined the impact of aphid populations on the incidence of *S. sclerotiorum* in the field. Additionally, we analyzed the effects of *S. sclerotiorum* on aphid feeding behavior in the laboratory using the electropenetrography (EPG) technique.

## 2. Materials and Methods

### 2.1. Plants

Two cultivars of *Brassica napus* var. *napus* (L.) were selected from the Laboratory of Plant Breeding of Anhui Academy of Agricultural Sciences, China: the spring oilseed rape cultivar “Xinyou17” (with Ogura cytoplasmic male sterility) and the winter oilseed rape cultivar “Zheping4” (bred through hybridization and systematic breeding from two autogamous inbred lines). According to Hao et al. [32], “Zheping4” relative impediments were found in the mesophyll and phloem, while “Xinyou17” relative impediments were located at the leaf surface level. The spring oilseed rape cultivar “Xinyou17” is grown in Northwest China and has been grown widely for a long time. The winter oilseed rape cultivar “Zheping4” is grown mainly in the middle and lower reaches of the Yangtze River in China. These two cultivars are often used as controls in production to test the traits of other cultivars.

The experiment utilized autoclaved pots and soil. Prior to planting, the seeds underwent surface sterilization with 10% (*v*/*v*) sodium hypochloride (Merck, Darmstadt, Germany) for one minute and 0.1% (*v*/*v*) Tween 20 (Sigma-Aldrich, Saint Louis, MO, USA) for one minute. The seeds were then washed in 96% ethanol (Guangzhou Jinhua Da Chemical Reagent Co., Guangzhou, China) and rinsed five times in sterile distilled water, following the method outlined by Boszoradova et al. [33]. The plants were grown in 13-cm diameter plastic pots containing a mixture of peat moss, vermiculite, organic fertilizer (N + P_2_O_5_ + K_2_O 2%, organic matter 40%, Zhongnuo, Huaian, China), and perlite (10:10:10:1 ratio). The plants were cultivated at a temperature of 25 ± 1 °C, 75 ± 5% relative humidity, and a 12:12 (light:dark) photoperiod. They were regularly watered but received no additional fertilizer. Each *B. napus* cultivar was inoculated with either SSR or a mock inoculation when the plants had two fully expanded leaves. A 5 cm diameter circle on a detached leaf of a four-week-old plant was spread with one gram of mycelia (wet weight) and incubated in a sealed and humidified tray at room temperature [34]. These treated plants were used for aphid vectoring SSR and electropenetrography (EPG) studies during the four-leaf stage, as per our previous research [35,36].

### 2.2. Aphids

*Brevicoryne brassicae*, commonly known as cabbage aphids, were obtained from oilseed rape plants growing in the greenhouse at the Institute of Vegetables, Zhejiang Academy of Agricultural Sciences. The aphids were reared in a controlled growth chamber for one year on a *Brassica oleracea* var. *capitata* (L.) (*Capparales: Brassicaceae*) cultivar at 25 ± 1 °C, 75 ± 5% relative humidity, and 16:8 (light:dark) photoperiod. All the aphids used in the study were descendants of a single virginoparous apterous aphid. Individual aphids from this colony were used in the experiments described below.

### 2.3. Determination of Host Preference by Y-Tube Olfactometer

To determine the behavioral response of aphids to *B. napus*, we conducted a series of Y-tube olfactometer behavioral bioassays, following the method of George et al. [37]. The odor source was provided by plants of two oilseed rape cultivars at the four-leaf stage, while clean air was used as a control. The Y-tube olfactometer consisted of colorless, transparent glass tubes with three arms, each measuring 10 cm in length and 2 cm in inner diameter. The angle between the two arms was 60°. Air was drawn through the activated carbon, the odor source bottle, and the flow meter into both arms using the atmospheric collector as an air pump. The airflow rate was 100 mL/min, the room temperature was maintained at 20 ± 1 °C, relative humidity was kept at 70 ± 5%, and the light intensity was 3.25 µmol m^−2^s^−1^. One single aphid was released at the halfway point of the glass tube. It was considered to be responsive when it reached an arm that was more than 5 cm away and vice versa. Each aphid was tested once, and the position of the two arms was exchanged every 5 aphids. Each treatment was conducted with 30 aphids, and the number of responding aphids was recorded. At the end of the test, the Y-tube olfactometer and the link hose were wiped with anhydrous ethanol. All treatments were performed five times.

### 2.4. Fungi

The *S. sclerotiorum* strain was kindly provided by Prof. Li Qiangsheng from the Laboratory of Oilseed Rape Pests of Anhui Academy of Agricultural Sciences, China. The isolate was chosen for its virulence and because it was obtained from a site with significant disease incidence. The culture was prepared using methods similar to those described by Qandah [38], with slight modifications.

*Sclerotinia sclerotiorum* fungal cultures were grown on Difco potato dextrose broth (Becton, Dickinson and Company, Franklin Lakes, NJ, USA) amended with 25 ppm ampicillin (MedChemExpress, Shanghai, China) and streptomycin (Sigma-Aldrich, Saint Louis, MO, USA) in 200-mL Erlenmeyer flasks. The cultures were grown in darkness at room temperature (approximately 23 °C) with gentle agitation for about 7 days until a large mass of mycelium had formed. The mycelium was washed with sterile water after decanting the supernatant [39]. Actively growing hyphae from these colonies were then transferred into dishes containing potato dextrose agar (PDA; Merck, Darmstadt, Germany) for sclerotia production. The PDA media was autoclaved for 20 min at 121 °C and 10^3^ KPa. After the media had cooled, multiple 5 mm-diameter agar plugs containing hyphal tips from the isolate were inoculated into the dishes. The inoculated PDA media was sealed and incubated at 21 °C for 2 weeks with a 12-h light/dark cycle. After incubation, the sclerotia was removed from the surface of the PDA media. To condition all the sclerotia for carpogenic germination, they underwent three successive cycles of 24 h of freeze at −20 °C followed by 24 h of thawing at 21 °C [38]. After the cycles, all sclerotia were disinfested by immersion in 5.76% NaOCl for 1 min. They were then rinsed three times in sterile distilled water (SDW) for 1 minute each time and lightly blotted on sterilized filter paper. Four disinfested sclerotia were half-buried in individual 9-cm diameter polystyrene Petri dishes containing 10 g of sterile white sand. The sand in each dish was moistened by adding 4.1 mL of SDW until saturation. The dishes were then sealed with Parafilm and incubated in a growth chamber for eight weeks, alternating between 12 °C/16 h of light and −18 °C/8 h of darkness. The freeze-thawed sclerotia germinated to form apothecia in the Petri dishes with white sand, and then the fully expanded apothecia were transferred to a new 5-cm dish containing a layer of water-saturated autoclaved white sand for ascospore collection. The lids were replaced every 1–2 days, and the ascospore-laden lids were stored in dry conditions at 4 °C until used. At the time of use, the ascospores were collected in vials by washing them off with 1 mL of SDW. Ascospore concentrations were estimated using a hemacytometer (Cambridge Scientific Instruments, Buffalo, NY, USA) and adjusted to a suspension of 1 × 10^6^ ascospores/mL. The suspension was then poured into a 50 mL plastic tube, following the methods described by Qandah [38].

### 2.5. Identification of S. sclerotiorum

To identify the pathogen, Prof. Fei Weixin, a plant pathologist, observed ascospore traits using microscopy and observed the colonies formed by cultivating ascospore-carrying aphids or diseased plant tissues on media.

### 2.6. Disease Assessment

The resistance was evaluated by inoculating the stem with inocula as described by Mei et al. [40,41] and Zhu [42] with minor modifications. The experiment was conducted in 2020–2021 using a complete randomized block design with ten replications in two disease nursery plots located in Anhui (Maanshan) (118°38′47″ E, 31°31′7″ N) and Qinghai (Xining) (101°45′24″ E, 36°45′56″ N), China. Twenty plants of each cultivar were planted in two rows, with 30 cm between rows and 25 cm within rows. Winter and spring-type oilseed rape cultivars were sown on two different dates: 1 October and 26 March. Field management followed regular breeding practices without the use of antifungal agents.

*Sclerotinia sclerotiorum* was cultured on potato dextrose agar (PDA) medium, which consisted of 20% potato, 2% dextrose, and 1.5% agar. Sterilized toothpicks were arranged radially in Petri dishes containing PDA medium and co-cultured with the fungus in the dark at 20 °C. For each test line row, ten plants were randomly selected and inoculated when 50% of the plants in the row had at least one opened flower [43]. The stems were pierced at a height of 20 cm above the ground using a 3-mm-diameter electric drill and then inserted with mycelium-covered toothpicks. Afterward, the inoculated stems were wrapped with Parafilm^®^ tape (Bemis Co., Oshkosh, WI, USA) to maintain moisture. Plants were irrigated once daily for 10 min using overhead sprinklers for three days and then only when natural rainfall was insufficient for robust disease development. Stem lesion length was measured using a ruler three weeks after inoculation. Resistance was tested in five to ten individuals from each plot. A registered rapeseed cultivar in China, “Zhongyou 821”, known for its partial resistance against *S. sclerotiorum*, was used as a resistant control [44]. The susceptibility of plants inoculated with *S. sclerotiorum* was measured three weeks after inoculation using lesion length on a scale of 0 to 4. A score of 0 indicated no visible symptoms, and a score of 1 indicated a lesion length of 1–2 cm, while a score of 2 indicated a lesion length of 2–3 cm, a score of 3 indicated a lesion length of 3–4 cm, and a score of 4 indicated a lesion length of more than 4 cm. The resistance of oilseed rape to *S. sclerotiorum* was evaluated using the relative resistance index (RRI) (Table 1).

Disease index DI (%) = Σ[number of diseased plants at every scale × representative value at every scale]/[Total number of investigated plants × representative value at the highest scale] × 100.

Relative resistance index (RRI) = ln[DIm/(100 − DIm)] − ln[DIck/(100 − DIck)] [42];

In the formula:

RRI—relative resistance index of identified cultivars;

DIm—disease index of identified cultivars;

DIck—disease index of the control cultivar “Zhongyou 821”;

The disease index and relative resistance index (Table 1) are rounded to one decimal place [42].

### 2.7. Relationship between Aphid Incidence and Incidence of S. sclerotiorum in the Field

Small-scale field plot experiments were conducted in a net house (10 × 4.5 m) over three years (2019–2021) to evaluate two cultivars. “Zheping4” was sown at Maanshan (118°38′47″ E, 31°31′7″ N), Anhui, and “Xinyou17” was sown at Xining (101°45′24″ E, 36°45′56″ N), Qinghai. The field sites had been used as *Sclerotinia* disease nurseries for several years to evaluate canola and had a history of *Sclerotinia* diseases. The experiment comprised field plots arranged in a randomized block design, with each cultivar and pesticide treatment replicated three times. Two pesticide treatments were applied: (a) control (no pesticide application) and (b) sprayed (pesticide application of Dingfeng^®^ (50% pymetrozine, Syngenta Nantong Crop Protection Co., Nantong, China) 0.27 kg/ha at the seedling stage). The plots were sown and managed according to recommended current farmer practices. Fertilizer was applied as required during the season to sustain high yields, but no fungicide was used. The aphids were placed on oilseed rape seedlings for approximately 5–7 days after the aphid population had been established on the oilseed rape and the next generation of oilseed rape aphids had been produced. During the flowering period of oilseed rape, the number of aphids was investigated separately in fields sprayed with insecticides and fields not sprayed with insecticides. In each field, ten points were sampled in parallel lines, and ten plants were continuously investigated in each point by rows [2]. The incidence of SSR in all experiments was recorded at the end of the growing season, approximately one week before harvest. One hundred randomly selected plants in each plot were assessed for disease presence. A plant was considered infected if the main stem or a branch was discolored or shredded, with sclerotia present [45]. Disease incidence for each plot was calculated as the percentage of plants with symptoms of SSR. Harvesting commenced approximately 30 days after final flowering when 2/3 of the plant’s pods were yellowish green, the pods at the base of the main inflorescences turned loquat-yellow, and the seed coat was blackish brown. The oilseed rape seeds were weighed after drying [46].

### 2.8. Determining If Aphids Could Transfer SSR from Symptomatic Oilseed Rape Seedlings to Healthy Oilseed Rape Seedlings in a Laboratory

#### 2.8.1. Influence of Aphid Feeding on the Incidence of *S. sclerotiorum*

Ten aphids were starved for 24 h and then placed in a clip-cage (bottom diameter × top opening diameter × depth 30 mm × 40 mm × 35 mm) fixed to the top leaf of oilseed rape for 48 h. After 48 h, the aphids were removed from the plant. Next, four droplets of ascospore suspension, each 1 mL in volume, were deposited on the leaf that had been fed on by the aphids using a micropipette. As a control, the plants were not fed on by aphids. After inoculation, the trays containing the plants were covered with a transparent dome to maintain humidity levels above 85%. The plants were then incubated in the dark at a temperature of 22 ± 1 °C and 100% relative humidity for 48 h. Following this, they were transferred to a greenhouse and incubated at a temperature of 18–21 °C. Observations were made once a week for four weeks. To confirm that observations made on inoculated plants were a response to invasion by *S. sclerotiorum*, inoculated control plants were used without following aphid feeding and maintained under the same conditions as the inoculated plants [43]. The treatments were performed five times.

#### 2.8.2. Ability of Aphids to Carry Ascospores of *S. sclerotiorum*

Ten cabbage aphids were placed in cups containing a soft oilseed rape leaf. The stem was wrapped with wet cotton. The insects were then sprayed with a spore suspension, as described by Al-Shindah et al. [47]. Ten aphids were removed after spraying and placed on oilseed rape plants with four leaves. Ten aphids were placed in a transparent plastic cylindrical cage (16 cm in diameter, 28 cm in height) covered with screen mesh (mesh size 0.1 mm × 0.1 mm) for ventilation per plant. The cage was kept in an incubator with a photoperiod (L:D) of 16:8 h at 22 ± 1 °C. After 72 h, the aphids were removed, and the plants were placed in darkness at 22 ± 1 °C and 100% relative humidity for two days to observe the development of SSR. Ten other aphids or diseased plant tissues were cultured directly on media to form colonies to confirm the presence of *S. sclerotiorum* by Prof. Fei Weixin. Each treatment was performed five times.

### 2.9. EPG Experiments

The experiment involved three treatments for each oilseed rape cultivar: control (aphids not carrying ascospores on uninfected plants), treatment I (aphids not carrying ascospores on infected plants), and treatment II (aphids with adherent ascospores on uninfected plants). All plants were used at the 4-leaf stage. Diseased plants and aphids carrying ascospores were treated as described above. Aphid feeding behavior was monitored in each treatment using the EPG method developed by Tjallingii [48]. EPG is a real-time monitoring technique used to study the interactions between plants and herbivorous insects. This method involves recording electrical signals generated as insects feed on plant tissues, providing valuable insights into insect feeding behaviors, feeding site selection, and plant responses to insect feeding. In the EPG experiments, aphids and plants need to be connected to form a closed circuit during aphid feeding. For this connection, a gold wire is glued to the aphid, and a copper electrode is inserted into the soil near the roots of a potted plant. When the aphid stylet penetrates the plant, a closed circuit is created [48]. Hao et al. [32] employed the EPG technique to track the penetration activities of apterous adult aphids on oilseed rape plants in a Faraday cage. The tethered aphid was placed on the upper side of the mature leaf midrib of the test plant within 30 min of being removed from the raising plant. Each treatment was recorded over 30 times in a laboratory setting with constant lighting and a temperature of 25 ± 1 °C. Aphids and plants were used only once for each recording. For each cultivar, three treatments were conducted, with over 30 replicates in each treatment. Each replicate consisted of one aphid and one plant, resulting in a total of 90 aphids and 90 plants. The feeding behavior of *B. brassicae* was monitored for 6 h. The waveform definitions recorded in EPG analyses for each aphid are listed in Table S1.

### 2.10. Statistical Analyses

The data were transformed to meet the assumptions of normality of residuals and homoscedasticity for analysis of variance (ANOVA). The normality of residuals was assessed using Shapiro–Wilk’s test, and homoscedasticity was assessed using Cochran’s test for outlying variance. Comparisons between different treatments and disease severity index were performed using ANOVA with either the Kruskal–Wallis test or Fisher’s least significant difference (LSD) and were considered significant at *p* ≤ 0.05 [43,49]. The EPG data were transformed using square-root transformation for the number of occurrences, natural log transformation for the duration, and square-root arcsine transformation for the proportion. This study investigates the relative acceptance of aphids and differences in response to *S. sclerotiorum* infection between the two oilseed rape cultivars. The differences in EPG variables were compared between control (feeding behavior of aphids not carrying ascospores on uninfected plants) and treatment I (feeding behavior of aphids not carrying ascospores on infected plants) and between control and treatment II (feeding behavior of aphids with adherent ascospores on uninfected plants) on the two cultivars. Non-Gaussian variables were analyzed using the Mann–Whitney *U*-test, while Gaussian variables were analyzed using Student’s *t*-test. The statistical analysis was conducted using SAS 9.2 software (SAS Institute Inc., Cary, NC, USA). Two-way ANOVA was used to analyze the main effects of cultivar and infection status, as well as their interaction. A significance level of *p* = 0.05 was chosen. Although all statistics were calculated on all variables, only the essential variables are presented in the tables and figures [32].

## 3. Results

### 3.1. Host Selection Studies

The results from the Y-tube olfactometer showed that the attractant rate of “Zheping4” (0.91 ± 0.04) to aphids was significantly higher than that (0.66 ± 0.04) of “Xinyou17” (F = 1.00, *p* = 0.0017).

### 3.2. Sclerotinia Disease Assessment

The resistance (susceptibility) of each oilseed rape cultivar to *S. sclerotiorum* was determined by artificial inoculation with the fungus, according to the criteria for classification of resistance levels.

Table 2 shows the relative resistance index for each of the three assessments with ten replications. The average relative resistance index of “Xinyou17” in three assessments was −0.07, indicating low resistance to *S. sclerotiorum*. In contrast, the average value of “Zheping4” in three tests was 0.42, indicating low susceptibility to *S. sclerotiorum*.

### 3.3. Relationship between Aphid Populations and the Incidence of S. sclerotiorum in the Field

The data from the three-year field trials showed that the insecticides effectively reduced the number of aphids and the incidence of *S. sclerotiorum* in both cultivars despite being planted in different locations (Table 3).

The correlation analysis revealed a highly significant positive correlation (*r* = 0.7929, *p* = 0.0021) between the number of aphids and the incidence of *S. sclerotiorum*. However, there was no significant correlation (*r* = 0.1130, *p* = 0.7265) between the thousand-seed weight and the incidence of *S. sclerotiorum*. There was no significant correlation (*r* = 0.1890, *p* = 0.5564) found between the number of aphids and the thousand-seed weight. Controlling aphids in seedlings cannot only prevent the direct damage caused by aphids but also reduce the damage caused by *S. sclerotiorum*.

Except for thousand-seed weight after insecticide application, there were significant interactions between environment and cultivar for all other variables, especially for aphid number with/without insecticide application and incidence of *S. sclerotiorum* with/without insecticide application, which were highly significant.

### 3.4. Influence of Aphid Feeding on the Development of S. sclerotiorum

#### 3.4.1. Influence of Aphid Feeding and Non-Feeding on the Incidence of *S. sclerotiorum*

**Figure 1:**
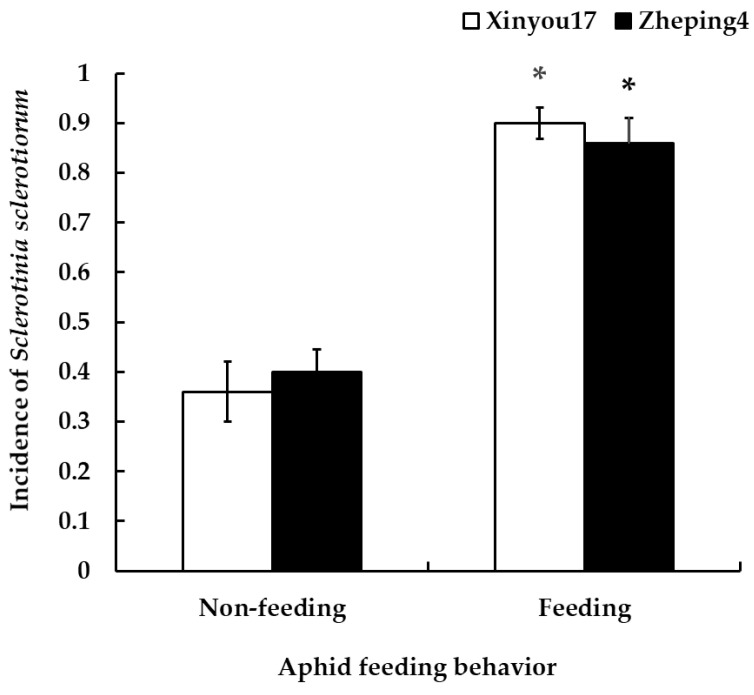
Influence of aphid feeding on the incidence of *Sclerotinia sclerotiorum* in the two oilseed rape cultivars “Xinyou17” and “Zheping4”. The values in the figure represent mean ± standard error (SE). The asterisk on the columns indicates a statistical difference between unfed plants and fed plants of the same cultivar.

Significant differences were found in the incidence of *S. sclerotiorum* on both cultivars after both aphid non-feeding and post-feeding plants. On “Xinyou17”, the incidence of *S. sclerotiorum* increased from 36% to 90% after aphid feeding (F = 2.75, *p* = 0.0005). Similarly, on “Zheping4”, the incidence of *S. sclerotiorum* increased from 40% to 86% after aphid feeding (F = 8.92, *p* = 0.0007). There was no significant difference in the incidence of *S. sclerotiorum* between the two cultivars, regardless of feeding (F = 3.81, *p* = 0.5450 for not feeding; F = 1.17, *p* = 0.5580 for feeding) (see Figure 1).

#### 3.4.2. Ability of Aphids to Transmit Ascospores

When aphids are sprayed with ascospores and then introduced to healthy oilseed rape plants, it can lead to *S. sclerotiorum* infection. The incidence of infection on both “Xinyou17” and “Zheping4” can reach up to 20% (0.24 ± 0.06 on “Xinyou17”; 0.20 ± 0.05 on “Zheping4”), with no significant difference between the two cultivars (F = 1.20, *p* = 0.6357).

### 3.5. Determination of Aphid Feeding Behavior by the EPG Technique

#### 3.5.1. Treatment I—Changes in the Feeding Behavior of Aphids in Plants Infected with *S. sclerotiorum*

**Figure 2:**
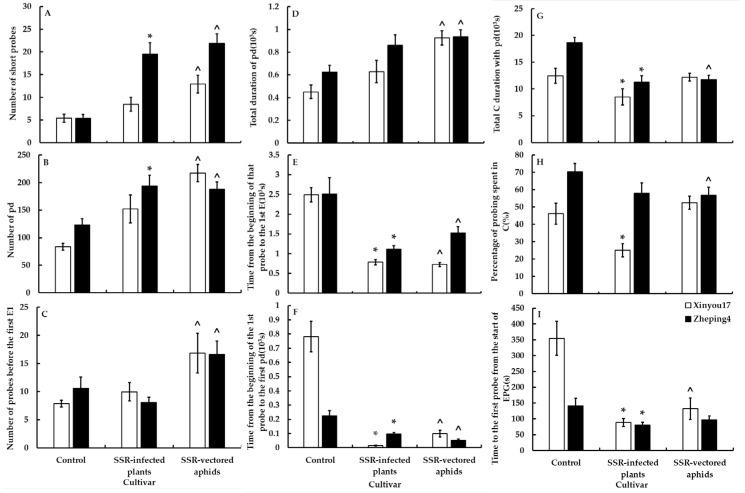
The surface-mesophyll-related variables of *Brevicoryne brassicae* probing behavior on the two oilseed rape cultivars in the *Sclerotinia sclerotiorum*-infected and uninfected treatments. The figure’s values display mean ± standard error (SE). Following the square-root transformation for frequency variables, natural log transformation for time variables, and square-arcsine for percentage variables, these data were compared using Student’s *t*-tests (for Gaussian variables) or Mann–Whitney *U*-tests (for non-Gaussian variables). It was decided to set the significance level at *p* < 0.05. The * on the columns denotes a statistical difference between the infected plant and control in the same cultivar; the ˆ on the columns indicates a statistical difference between aphids with ascospores and control in the same cultivar. (**A**): Number of short probes; (**B**): Number of pd; (**C**): Number of probes before the first E1; (**D**): Total duration of pd; (**E**): Time from the beginning of that probe to the first E; (**F**): Time from the beginning of the first probe to the first pd; (**G**): Total C duration with pd; (**H**): Percentage of probing spent in C; (**I**): Time to the first probe from the start of EPG.

**Figure 3:**
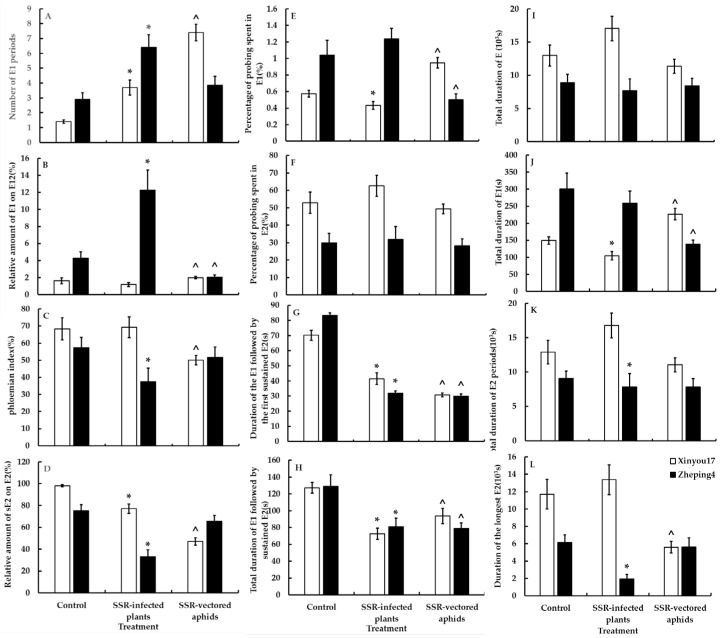
The phloem-related variables of *Brevicoryne brassicae* probing behavior on the two oilseed rape cultivars in the *Sclerotinia sclerotiorum*-infected and uninfected treatments. The figure’s values display mean ± standard error (SE). Following the square-root transformation for frequency variables, natural log transformation for time variables, and square-arcsine for percentage variables, these data were compared using Student’s *t*-tests (for Gaussian variables) or Mann–Whitney *U*-tests (for non-Gaussian variables). It was decided to set the significance level at *p* < 0.05. The * on the columns denotes a statistical difference between the infected plant and control in the same cultivar; the ˆ on the columns indicates a statistical difference between aphids with ascospores and control in the same cultivar. (**A**): Number of E1 periods; (**B**): Relative amount of E1 on E12; (**C**): Percentage of the time of the E2 after the start of the first E2 (Phloemian index); (**D**): Relative amount of sE2 on E2; (**E**): Percentage of probing spent in E1; (**F**): Percentage of probing spent in E2; (**G**): Duration of the E1 followed by the first sustained E2; (**H**): Total duration of E1 followed by sustained E2; (**I**): Total duration of the E phases; (**J**): Total duration of E1; (**K**): Total duration of E2 periods; (**L**): Duration of the longest E2.

Figure 2 and Figure 3 show that aphid feeding behavior was significantly altered in all leaf tissue levels after plants were infected with *S. sclerotiorum*. Following infection of oilseed rape with *S. sclerotiorum*, aphids initiated their first puncture significantly sooner than in oilseed rape not infected with *S. sclerotiorum* (F = 1.12, *p* < 0.0001 on “Xinyou17”; F = 1.46, *p* = 0.0102 on “Zheping4”). In the mesophyll, aphids began probing the mesophyll cells earlier (U = 1, *p* < 0.0001 on “Xinyou17”; U = 53, *p* = 0.0001 on “Zheping4”) and had a significantly shorter pathway duration (F = 1.69, *p* = 0.0302 on “Xinyou17”; U = 45, *p* < 0.0001 on “Zheping4”). In the phloem, aphids secreted saliva more frequently (U = 50, *p* < 0.0001 on “Xinyou17”; F = 1.55, *p* = 0.0009 on “Zheping4”) but reduced salivation prior to feeding and ingestion. On “Xinyou17”, which has low resistance to *S. sclerotiorum*, the pathway period was shortened, but there was no significant change in the frequency and duration of puncturing the mesophyll cells. The frequency of salivary secretion increased, but there was no significant change in the overall salivary secretion or the percentage of ingestion. Regarding “Zheping4”, which is low susceptible to *S. sclerotiorum*, the frequency of aphids probing mesophyll cells (F = 1.60, *p* = 0.0054) and brief probing increased significantly (U = 57, *p* = 0.0001) after infection with *S. sclerotiorum*. Additionally, the percentage of saliva secretion in the phloem increased significantly (U = 46, *p* < 0.0001), while the percentage of feeding decreased significantly (F = 1.69, *p* = 0.0482).

#### 3.5.2. Treatment II—The Effect of Ascospores on the Feeding Behavior of Aphids

There were notable differences in the feeding behavior of aphids carrying ascospores compared to those that did not. Aphids carrying ascospores spent less time per probe and more time non-probing (U = 106, *p* = 0.0114 on “Xinyou17”; U = 11, *p* < 0.0001 on “Zheping4”). In the mesophyll, aphids carrying ascospores began puncturing cells more quickly (F = 1.05, *p* < 0.0001 on “Xinyou17”; F = 1.38, *p* < 0.0001 on “Zheping4”) and increased the frequency and duration of short probes as well as cell punctures, with shorter pathway durations. In the phloem, aphids carrying ascospores exhibited reduced salivation prior to feeding. The feeding behavior of aphids carrying ascospores differed between the two cultivars. On “Xinyou17”, aphids carrying ascospores began penetrating leaves more quickly (F = 1.11, *p* = 0.0001), but on “Zheping4”, they did not differ significantly from aphids not carrying ascospores. Aphids carrying ascospores increased total salivary secretion in the phloem of “Xinyou17” (F = 1.17, *p* = 0.0005) but decreased it in the phloem of “Zheping4” (U = 96, *p* = 0.0051). There was a significant decrease in phloem feeding by aphids carrying ascospores on “Xinyou17” compared to those not carrying ascospores (F = 1.38, *p* < 0.0001), as shown in Table S2. However, there was no significant change in feeding on “Zheping4” (Figure 2 and Figure 3, Table S2).

#### 3.5.3. Analysis of Cultivar and *S. sclerotiorum* Infection Main Effects

The timing of aphids began their first penetration on the leaf surface in the epidermis-mesophyll variables of plants infected with *S. sclerotiorum* was influenced by both cultivar and *S. sclerotiorum* infection. The cross-effects of cultivar and *S. sclerotiorum* infection also reached a significant level. In the mesophyll, the number of short probes was significantly affected by the cultivar, *S. sclerotiorum* infection, and the interaction between the two factors. The timing of aphids’ beginning mesophyll cell probing was significantly influenced by cultivar and *S. sclerotiorum* infection, as well as their interaction. Both cultivar and *S. sclerotiorum* infection also affected the frequency and periods of cell puncture, but their interaction did not have a significant effect. Both cultivar and *S. sclerotiorum* infection had a similar effect on the pathway duration. However, the cross-effects of the two were not significant, as shown in Table 4.

In the phloem, salivary secretion frequency and duration were significantly influenced by cultivar and *S. sclerotiorum* infection but not by the interaction of the two. Salivary secretion before the first feeding was primarily affected by *S. sclerotiorum* infection and the interaction of cultivar and *S. sclerotiorum* infection. The cultivar and cross-effects between cultivar and *S. sclerotiorum* infection significantly affected the proportion of salivary secretion. Meanwhile, cultivars had the main influence on feeding variables.

The time taken for aphids carrying ascospores to first penetrate the leaf surface was influenced by cultivar, *S. sclerotiorum* infection, and their interaction. The frequency of short probes of aphids carrying ascospores in the mesophyll was also influenced by cultivar, *S. sclerotiorum* infection, and their interaction. Additionally, the time at which aphids started their first cell puncture was also significantly affected by cultivar and *S. sclerotiorum* infection. In contrast, *S. sclerotiorum* infection had a greater impact on intracellular penetration, while cultivar, *S. sclerotiorum* infection, and their interaction affected pathway duration. For aphids carrying ascospores in the phloem, *S. sclerotiorum* infection and the interaction between cultivar and *S. sclerotiorum* infection affected the frequency of salivation and salivation before ingestion. Ingestion, on the other hand, was mainly influenced by cultivar or *S. sclerotiorum* infection (see Table 4).

## 4. Discussion

### 4.1. Differences in Resistance between the Two Cultivars

The study utilized two oilseed rape cultivars, namely “Xinyou17” (spring type) and “Zheping4” (winter type). The Y-tube olfactometer and EPG results [50] indicated that the leaf surface of winter oilseed rape cultivar “Zheping4” was more attractive to aphids than “Xinyou17”. Although the leaf surface of “Xinyou17” was more obstructive to aphids (aphids took longer to start their first probe), it was more susceptible to aphids in both the mesophyll and phloem, as shown by the fact that aphids in the “Xinyou17” with less intracellular penetration and a shorter pathway time in the mesophyll, and longer probing in the phloem, less frequent salivation, shorter salivation, shorter salivation before the first ingestion, and longer ingestion.

Following artificial inoculation with *S. sclerotiorum* to assess the resistance of the two cultivars, the results indicated that “Xinyou17” exhibited low resistance to *S. sclerotiorum*, while “Zheping4” was low susceptible to the fungus. For the disease to take hold, the mechanical forces generated by the appressorium aid in the penetration of the host tissue by the fungal pathogen, which then secretes enzymes to degrade the host cell wall, making the penetration process easier for the pathogen. Plants benefit from the thick and tough walls of epidermal cells, which make it difficult for pathogens to penetrate directly and help plants resist invaders [51]. According to Hao et al. [32], “Xinyou17” has a thicker upper epidermis and fewer trichomes than “Zheping4”. Compared to “Zheping4”, the leaf surface of “Xinyou17” does not attract aphids and may impede aphid probing behavior and pathogen infection due to its thick epidermis. This may be associated with its low resistance to *S. sclerotiorum*.

### 4.2. The Relationship between Aphids and the Occurrence of S. sclerotiorum

Field trials were conducted in suitable planting areas based on the characteristics of oilseed rape. Both spring oilseed rape “Xinyou17” and winter oilseed rape “Zheping4” showed a correlation between a higher aphid population and increased occurrence of *S. sclerotiorum*. The incidence of *S. sclerotiorum* was reduced after the aphid population was controlled with insecticides. The correlation analysis showed a significant positive correlation between the number of aphids and the incidence of *S. sclerotiorum*. This suggests that aphids and *S. sclerotiorum* have an interplay rather than existing as two unrelated individuals. Similarly, the prevalence of pecky rice was dependent on the abundance of fungal and *Oebalus pugnax* (Fabricius) (*Hemiptera: Pentatomidae*), with a greater proportion of pecks found in fields infected with substantial quantities of *Helminthosporium oryzae* (Breda de Haan) as opposed to fields infested with high concentrations of *O. pugnax*. In contrast, fields with high *H. oryzae* infection rates and high *O. pugnax* densities had the greatest percentage of peck damage. The presence of *O. pugnax* increases the incidence of peckiness (as cited in [52]). Douglas and Tullis [53] observed an increased occurrence of peckiness in the presence of fungal interaction with *O. pugnax* during the invasion, as well as in the case of empty kernels. *Fusarium oxysporum* var. *redolens* (Wollenw.) Gordon is commonly regarded as a wound pathogen of maize (*Zea mays* L.) [54,55]. This implies that the significance of its contribution to root disease may increase in instances where plants are harmed by root-feeding insects or nematodes. In both field and laboratory experiments, a greater percentage of plants that were cultivated from kernels treated with *Fusarium* displayed leaf-feeding damage inflicted by the western spotted cucumber beetle (*Diabrotica undecimpunctata undecimpunctata* (Mannerheim) (*Coleoptera: Chrysomelidae*), WSCB) compared to plants grown from disinfested kernels. The subsequent larval damage to the root systems may allow *Fusarium* to enter the vascular system of the roots. The WSCB can likely vector pathogenic *Fusarium* species [56].

In our laboratory trials, we observed a significant increase in the incidence of *S. sclerotiorum* following aphid feeding. Additionally, we found that aphids were able to carry ascospores after being sprayed with ascospore suspensions and spread them to healthy plants, causing disease. Although the incidence of aphid-induced *S. sclerotiorum* was low, the possibility of introduction of *S. sclerotiorum* infection due to improper experimental practices existed, and the direct evidence was lacking in our study, it could be shown that aphids do indeed promote *S. sclerotiorum* infection. Insect herbivores are commonly associated with fungal, bacterial, and viral microbiomes. These microbiomes can be located externally on the insect’s exoskeleton or within the insect’s gut, body, salivary glands, or between insect cells [57,58,59,60]. Associations with fungi are only possible through external-body attachment, such as in the case of acaro-pathogenic fungi [61]. Yamoah [16] investigated the ability of four phytophagous insects: *Apion ulicis* (Förster) (*Coleoptera: Apionidae*), *Cydia ulicetana* (Denis and Schiffermüller) (*Lepidoptera: Tortricidae*), *Epiphyas postvittana* (Walker) (*Lepidoptera: Tortricidae*), and *Sericothrips staphylinus* (Haliday) (*Thysanoptera: Thripidae*) on *Ulex europaeus* (L.) (*Fabaceae*) carrying and depositing *Fusarium tumidum* (Sherbakoff) conidia. After exposing the insects to *F. tumidum* for 24 h, many conidia were recovered. Each adult *E. postvittana* was found to be carrying 5000 *F. tumidum* conidia. When the insects carrying the conidia were placed on healthy plants, an average of 310 conidia were deposited per insect. Shamshad and colleagues [62] have provided documentation on the transmission of a recurrent disease known as dry bubble disease in mushroom farming. This disease is caused by *Lecanicillium fungicola* (Preuss), Zare, and Gams and is vectorized by *Lycoriella ingenua* (Dufour) (*Diptera: Sciaridae*). Using scanning electron microscopy (SEM), it has been consistently observed that fungal spores are present exclusively on the femorotibial joint setae and tarsi of all flies introduced onto culture plates containing *Trichoderma aggressivum* ft. *aggressivum* (Samuels and Gams). A small number of spores have also been detected on the ovipositors. It has been observed that female sciarid flies are attracted to mushroom compost that is infected with *T. aggressivum* rather than compost that is free of infection [63]. Upon release into the chamber, the flies have shown a preference for visiting Petri plates that have been inoculated with *T. aggressivum*. The spores adhere to the exoskeleton of the flies upon landing, either through passive or active harvesting. As a result, the flies subsequently visit clean Petri dishes, leading to infections of green mold [64]. Ascospores of *S. sclerotiorum* are covered in sticky mucilage, aiding adhesion to the substrates. Both senescent flower petals and wounded tissues have the potential to function as a highly effective external nutrient reservoir, facilitating the process of ascospore germination [38]. Therefore, it can be hypothesized that fungal spores may also adhere to the body surface of aphids and be transmitted to healthy plants.

### 4.3. Effect of S. sclerotiorum-Infected Plants on the Feeding Behavior of Aphids

Observing changes in aphid feeding behavior under the influence of *S. sclerotiorum* using EPG, it was found that infection of *S. sclerotiorum* on plants affected aphid feeding behavior at all tissue levels of plant leaves. The time taken by aphids to initiate their first puncture on *S. sclerotiorum*-infected oilseed rape plants was significantly shorter compared to uninfected oilseed rape. This suggests that *S. sclerotiorum* infection made the leaf surface of the plant less resistant or more attractive to aphids. Similar instances of increased attraction of plants to insects following fungal infection have been observed in several other fungal-insect mutualism systems. For instance, in the study of Braun et al. [65], female *Bradysia impatiens* were found to be more attracted to geranium seedlings infected with several plant-pathogenic *Pythium* spp. than to healthy seedlings. Similarly, McLeod et al. [66] reported that the bark beetle, *Hylurgopinus rufipes* (Eichhoff) (*Coleoptera: Scolytidae*), showed a preference for elm trees infected with the Dutch elm disease pathogen (*Ophiostoma novo-ulmi* Brasier) over uninfected elms. Pathogens that can be spread by insects may reap advantages from developing mechanisms that serve to entice insect vectors, leading to increased attraction of beetles to infected hosts and potentially increasing disease transmission and the abundance of infective vectors [67,68]. Certain fungi that are pathogenic to plants can generate chemical attractants, also known as volatiles, and food sources, such as sugars. These substances serve as stimuli that guide the transmission of spores by insects to hosts that have not yet been infected (as cited in [17]). *Ambrosiella xylebori* (Brader) is a primary symbiotic fungus that is typically transported in the mycetangium of *Xylosandrus compactus* (Eichhoff) (*Coleoptera: Scolytidae*) and sustains the growth of insects within the host tree. All documented *Ambrosiella* species generate a fragrant aroma [69], and these chemical volatiles may have a significant influence in attracting ambrosia beetles within the galleries [70]. *Fusarium verticillioides* (Saccardo) Nirenberg produces volatile compounds, such as acetaldehyde, ethyl acetate, and some alcohols that have been shown to attract sap beetles (*Coleoptera: Nitidulidae*) [71]. Conversely, clover root borers have been observed to be drawn towards diseased clover roots but not towards isolates of *Fusarium* species that induce root rot in clover [72]. This suggests that in certain scenarios, the volatiles emitted by plants experiencing stress may assume a more prominent role. Additional research is required to establish whether the responsible party is the volatiles emitted by the fungus, the volatiles released by the fungus-infected plants, or a combination of both.

Plant pathogens not only modify volatiles emitted by their hosts [66,73], but they can also cause changes in plant phloem, xylem, phytohormones, or micro-element ratios [74]. Our study found that aphids began intracellular penetration earlier and significantly shortened the pathway duration in the mesophyll infected with *S. sclerotiorum*. The changes mentioned in the text are similar to those caused by turnip mosaic virus (TuMV) infection. However, since aphids are vectors of TuMV transmission and acquire the virus through cell puncture [32], they should not acquire the fungus through cell puncture. *Sclerotinia sclerotiorum* infection of plants is suggested to alter the mesophyll structure [75], perhaps reducing its aphid resistance.

In the case of *B. napus*, its defense mechanism restricts the development and spread of *S. sclerotiorum* by producing antibiotic substances that inhibit its growth, such as the production of glucanase and chitinase enzymes facilitates the breakdown of fungal cell walls, the promotion of indolic GSL biosynthesis, and the generation of supplementary defensive enzymes acts as a hindrance to the virulence factors of *S. sclerotiorum* [76]. *Sclerotinia sclerotiorum* employs a diverse range of factors and complex strategies to establish disease and complete host plant infection. This includes releasing a combination of hydrolytic enzymes, detoxification systems, and effector proteins to disrupt the cell wall and host plant tissue, as well as nutrients to facilitate spread [34]. After colonizing the plant, *S. sclerotiorum* induces the biosynthesis of both glucosinolates (GSLs) and indolic GSLs, which are associated with *S. sclerotiorum* resistance [76,77,78]. GSLs hold a significant position as secondary metabolites in cruciferous vegetables and exhibit a close association with both biotic and abiotic stress factors. Several studies have shown that the content of GSLs is positively related to resistance against diseases and insects [79,80,81,82,83,84]. When a plant cell is ruptured due to pest or pathogen attack or mechanical wounding, the GSLs and myrosinase (hydrolytic enzymes, M) interact and undergo hydrolysis in the presence of water to liberate diverse substances, such as isothiocyanates (ITCs) [85], which possess a broad spectrum of biocidal properties [86,87]. Despite this, the ability of *S. sclerotiorum* to infect *Brassica* tissues persists. It has been observed that *S. sclerotiorum* can acquire tolerance to ITCs following initial exposure to sub-lethal doses [88]. *Sclerotinia sclerotiorum* also produces both exo-PGs (such as ssxpg1 and ssxpg2) and endo-PGs (such as sspg1, sspg5, and sspg6) [89]. Exo-PG enzymes are responsible for the hydrolysis of monomeric or dimeric glycosyl moieties present in the pectic cell wall polysaccharides, leading to the fragmentation of the substrate and the subsequent liberation of potential nutrients. Endo-PG catalyzes the hydrolysis of homogalacturonan [90]. Our study found that in the infected phloem, aphids secreted saliva more frequently but reduced salivation prior to ingestion and feeding. It is possible that *S. sclerotiorum* infection of the plants produces a substance, such as ITCs, that is unfavorable to aphid feeding. This may result in an increased frequency of salivary secretion but reduced feeding. Reduced aphid feeding also prevents competition for nutrients with *S. sclerotiorum*. *Ophiostomatoid* (blue-stain fungal) rely on being carried on the surface of the body and in the gut of dispersing beetles, *Ips typographus* (L.) (*Coleoptera: Curculionidae: Scolytinae*), before being introduced into breeding galleries in the phloem tissue of Norway spruce, *Picea abies* (L.) Karst. (*Pinales: Pinaceae*). According to Krokene et al. [91], Franceschi et al. [92], Lieutier et al. [93], Kirisits [94], Krokene [95], and Netherer and Hammerbacher [96], these fungi can assist beetles in overcoming and exhausting host defenses, induce the tree to establish hypersensitive wound reaction zones around infection sites, enclose areas of necrotic phloem where terpenes and phenolics accumulate, and ultimately cause the death of the tree. Furthermore, an increase in terpene in response to fungal inoculation greatly reduced future spruce bark beetle colonization in field studies with young Norway spruce [97]. It is unlikely that consumptive fungi provide direct nutritional benefits for beetle larval development [98] when *I. typographus* vectors *Ceratocystis polonica* (Siemaszko) C. Moreau. Because they did not observe an increase in phloem resource concentrations, such as nitrogen, following fungal inoculation, that would support this.

### 4.4. Changes in the Feeding Behavior of Aphids Carrying Ascospores

Invasive insects can carry a variety of fungal associates, which can indirectly affect insects through infected host plants. In some cases, fungi can have intimate interactions with their insect vectors [99,100]. Additionally, *S. sclerotiorum* can directly affect aphids through spores, altering their feeding behavior. The study found that aphids carrying *S. sclerotiorum* ascospores spent significantly less time probing and significantly more time not probing. When an insect attacks a plant, the plant undergoes cell wall modification, which involves the receptors activating the plant’s immune system to receive signals released by the insect. This is the plant’s initial chemical response to an insect attack [101]. The activation of plant immunity may impede aphid feeding behavior.

Following herbivore attack, various alterations take place in the plasma membrane of plant cells. Plant cell surface-localized pattern recognition receptors (PRRs) enhance plant immunity by recognizing plant-derived damage-associated molecular patterns (DAMPs), microbe-associated molecular patterns (MAMPs) [102], herbivore-associated molecular patterns (HAMPs) induced by chemical cues in herbivore oral secretions (OSs) or oviposition fluid [103], and phytocytokines, and activating PTI against pathogens [102]. HAMP compounds trigger the release of leaf volatiles and terpenoids [104]. Herbivore attacks cause changes in plasma membrane potential (Vm), followed by the generation of secondary messengers such as [Ca^2+^] cyt and reactive oxygen species (ROS) [103], as well as a rapid increase in phytohormones like JAs [105]. In the mesophyll, aphids carrying ascospores puncture cells more quickly, increasing the frequency and duration of short probes and cell punctures with shorter pathway durations. The increased obstructions are implied by faster and more frequent penetration of the mesophyll cells by aphids [106]. These changes resemble the effects of TuMV infection on aphid feeding behavior. However, since aphids are vectors of TuMV transmission and the virus-carrying aphids inoculate the virus through cell puncture [32], it is unlikely that the aphids carrying ascospores inoculate the fungus through cell puncture. Instead, the aphids may puncture the cells to release the necessary nutrients and create a suitable environment for the germination of ascospores. The ascospores actively guide the aphids to change their feeding behavior, creating conditions for their own propagation and development. This is advantageous for the success of *S. sclerotiorum* in colonizing and developing.

Plants accumulate secondary metabolites (SMs), such as phenolic compounds and tannins, following herbivore attacks [107,108,109,110]. Phenolic compounds, including lignin, coumarins, furanocoumarins, flavonoids, and tannins, act as feeding deterrents and toxins, reducing the nutritional value of plant food [111,112]. In this study, aphids carrying ascospores exhibited reduced salivation before feeding and decreased ingestion in the phloem. Lahr and Krokene [98] discovered that bark beetles and fungi have a mutualistic relationship. However, beetles may compete with fungi for food resources within the tree if they ingest phloem tissue before it is colonized by fungi. Therefore, aphids carrying ascospores may reduce feeding, which could help avoid competition for plant nutrients with *S. sclerotiorum* infection.

### 4.5. Relationship between Aphids and S. sclerotiorum and the Effect of Oilseed Rape Cultivars

Furthermore, the impact of *S. sclerotiorum* on aphid feeding behavior was affected by the cultivar of oilseed rape. A two-way ANOVA revealed that most variables were influenced by both the cultivar and *S. sclerotiorum* infection. Due to inherent differences in aphid and *S. sclerotiorum* resistance between “Xinyou17” and “Zheping4”, the effect of cultivar on the relationship between aphids and *S. sclerotiorum* can manifest itself in different ways. In cases where aphid feeding behavior was indirectly affected by *S. sclerotiorum* through plant infection, plant resistance to *S. sclerotiorum* was found to be more significant than plant resistance to aphids for this effect. In studies where *S. sclerotiorum* spores directly affected aphid feeding behavior, plant resistance to aphids was more significant than plant resistance to *S. sclerotiorum*. In general, *S. sclerotiorum* regulated aphid feeding behavior in a way that favored its own spread, regardless of the cultivar.

The relationships between fungi and their insect vectors can be roughly classified as mutualism [113,114,115], commensalism, and antagonism [116,117]. The relationships between invasive insects and their fungal companions can profoundly impact behavior, reproductive outcomes, population dynamics, and evolutionary processes [118]. Our studies suggest that aphid feeding behavior plays a crucial role in the spread of *S. sclerotiorum*. However, aphids do not appear to benefit from this process of transmission. Insect–microbial mutualisms often involve the microbe providing nutrition in exchange for the host insect’s protected environment [119], synthesizing various compounds and small molecules used by insects in social interactions, increasing fitness in extreme abiotic environments, overwhelming plant defenses, and providing protection from natural enemies (as cited in [120]). There are also several examples in which fungi appear to deceive insects (as cited in [121]). These fungi use olfactory and gustatory cues to attract insects to spore-rich areas. Subsequently, the spores adhere to the insect’s body and become disseminated as the insect traverses the terrain, but the enticed insects do not gain a nectar reward or any prospective fitness advantages through this spore dispersal mechanism. Therefore, this is not a mutually beneficial relationship. In some insect vectors for various phytopathogenic fungi, researchers have observed reduced survival, fecundity, biomass, and slower development [122,123,124]. In our study, we found that *S. sclerotiorum* infection inhibited plant resistance to aphids but also reduced aphid feeding. It is unclear from our current results whether aphid development was facilitated by improved access to nutrients after *S. sclerotiorum* infection. Further research is needed to confirm the interactions between aphids and *S. sclerotiorum*.

## 5. Conclusions

There appears to be an interaction between aphids and *S. sclerotiorum*. It has been indirectly demonstrated that aphids can carry and deposit *S. sclerotiorum* ascospores onto healthy plants, resulting in the development of *S. sclerotiorum*. The incidence of *S. sclerotiorum* increases after aphid feeding. However, direct evidence supporting this hypothesis is currently lacking.

*Sclerotinia sclerotiorum* can influence aphid probing behavior either directly, by being carried by aphids, or indirectly, by infecting the host plant. This influence is directional and varies in a way that favors the spread of *S. sclerotiorum*. This is consistent with observations in the field. By strengthening field monitoring, particularly for aphid occurrence during the seedling stage of oilseed rape, and paying timely attention to the disease susceptibility of oilseed rape petals in the field, we can comprehensively grasp the dynamics of oilseed rape pests and diseases in the field. This will enable us to determine the most appropriate control period.

## Figures and Tables

**Table 1:** Identification standard of oilseed rape resistance to *Sclerotinia sclerotiorum*.

Relative Resistant Index (RRI)	Evaluation of Resistance
RRI ≤ −1.2	High resistance (HR)
−1.2< RRI ≤ −0.7	Medium resistance (MR)
−0.7 < RRI ≤ 0	Low resistance (LR)
0 < RRI ≤ 0.9	Low susceptibility (LS)
0.9 < RRI ≤ 2.0	Medium susceptibility (MS)
RRI > 2.0	High susceptibility (HS)

**Table 2:** Artificial inoculation results of *Sclerotinia sclerotiorum*.

Cultivar	Relative Resistant Index (RRI)			Evaluation of Resistance
	1	2	3	
Xinyou17	−0.01	−0.12	−0.08	LR
Zheping4	0.43	0.41	0.42	LS

**Table 3:** Relationship between aphid infestation and the development of *Sclerotinia sclerotiorum* in the field.

Cultivar	Number of Aphids/100 Plants	Incidence of *S. sclerotiorum* %/100 Plants	Thousand-Seed Weight (g)	Number of Aphids after Insecticide Application/100 Plants	Incidence of *S. sclerotiorum* Following Insecticide Application %/100 Plants	Thousand-Seed Weight after Insecticide Application (g)
2019						
Xinyou17	875.86	15.44	3.16	32.45	5.51	3.30
Zheping4	5894.40	42.04	3.29	288.97	31.27	3.30
2020						
Xinyou17	996.67	22.15	3.05	14.76	4.39	3.11
Zheping4	2392.86	25.74	3.29	8.10	15.26	3.32
2021						
Xinyou17	220.00	13.50	3.13	11.43	4.16	3.18
Zheping4	3168.10	37.74	3.10	13.81	21.71	3.06
Cultivar × Environment Interaction	*p* < 0.0001	*p* < 0.0001	*p* = 0.0485	*p* < 0.0001	*p* < 0.0001	*p* = 0.6061

**Table 4:** The main effect analysis of major EPG variables in different levels of leaves.

Variable	Treatment ^1^	Cultivar Main Effects			*Sclerotinia sclerotiorum* Infection Main Effects			Cultivar × *S. sclerotiorum* Infection Interaction		
		F	*p*	Partial η^2^	F	*p*	Partial η^2^	F	*p*	Partial η^2^
Surface-mesophyll (Leaf)										
Time to the first probe from the start of EPG	Plant	8.71	**0.0042**	0.1028	36.96	**0.0000**	0.3272	6.23	**0.0147**	0.0758
	Aphid	8.87	**0.0039**	0.1045	21.82	**0.0000**	0.2231	6.47	**0.0130**	0.0785
Number of short probes	Plant	10.29	**0.0020**	0.1193	23.04	**0.0000**	0.2326	9.77	**0.0025**	0.1139
	Aphid	7.35	**0.0083**	0.0882	69.44	**0.0000**	0.4775	6.84	**0.0108**	0.0825
Time from the beginning of the first probe to the first pd	Plant	5.04	**0.0277**	0.0622	163.83	**0.0000**	0.6831	85.28	**0.0000**	0.5288
	Aphid	15.06	**0.0002**	0.1654	66.93	**0.0000**	0.4683	2.13	0.1484	0.0273
Number of pd	Plant	7.21	**0.0089**	0.0867	13.78	**0.0004**	0.1535	0.02	0.8901	0.0003
	Aphid	0.62	0.4343	0.0081	66.98	**0.0000**	0.4685	7.86	**0.0064**	0.0937
Total duration of pd	Plant	8.00	**0.0060**	0.0952	5.15	**0.0261**	0.0635	0.00	0.9845	0.0000
	Aphid	3.28	0.0742	0.0413	34.69	**0.0000**	0.3134	2.91	0.0921	0.0369
Time from the beginning of that probe to the first E	Plant	1.24	0.2699	0.0160	80.21	**0.0000**	0.5135	6.07	**0.0160**	0.0740
	Aphid	6.71	**0.0115**	0.0812	58.13	**0.0000**	0.4334	15.09	**0.0002**	0.1657
Total C duration with pd	Plant	13.88	**0.0004**	0.1544	17.34	**0.0001**	0.1857	0.06	0.8037	0.0008
	Aphid	7.35	**0.0083**	0.0882	4.29	**0.0418**	0.0534	10.55	**0.0017**	0.1219
Percentage of probing spent in C	Plant	28.36	**0.0000**	0.2718	10.76	**0.0016**	0.1240	0.55	0.4615	0.0072
	Aphid	8.30	**0.0052**	0.0984	0.89	0.3488	0.0116	4.35	**0.0403**	0.0542
Phloem										
Number of E1 periods	Plant	13.63	**0.0004**	0.1521	31.74	**0.0000**	0.2946	0.30	0.5836	0.0040
	Aphid	3.16	0.0794	0.0399	47.67	**0.0000**	0.3855	25.90	**0.0000**	0.2541
Total duration of E1	Plant	26.94	**0.0000**	0.2617	4.65	**0.0342**	0.0576	1.52	0.2221	0.0195
	Aphid	0.02	0.8795	0.0003	0.97	0.3287	0.0126	24.35	**0.0000**	0.2426
Duration of the E1 followed by the first sustained E2	Plant	0.05	0.8238	0.0007	177.64	**0.0000**	0.7004	11.72	**0.0010**	0.1336
	Aphid	3.50	0.0654	0.0440	495.09	**0.0000**	0.8669	6.90	**0.0104**	0.0833
Relative amount of E1 on E12	Plant	38.49	**0.0000**	0.3362	4.59	**0.0354**	0.0569	8.91	**0.0038**	0.1050
	Aphid	10.24	**0.0020**	0.1187	2.17	0.1448	0.0278	10.99	**0.0014**	0.1263
Percentage of probing spent in E1	Plant	28.05	**0.0000**	0.2695	0.04	0.8423	0.0005	4.34	**0.0405**	0.0541
	Aphid	0.81	0.3706	0.0106	0.30	0.5853	0.0039	17.88	**0.0001**	0.1905
Total duration of E2 periods	Plant	14.03	**0.0003**	0.1558	1.21	0.2749	0.0157	6.72	**0.0114**	0.0812
	Aphid	5.33	**0.0237**	0.0655	0.73	0.3969	0.0095	0.67	0.4158	0.0087
Duration of the longest E2	Plant	46.58	**0.0000**	0.3800	11.34	**0.0012**	0.1298	17.37	**0.0001**	0.1861
	Aphid	4.66	**0.0340**	0.0578	5.50	**0.0216**	0.0675	0.84	0.3622	0.0109
phloemian index: percentage of the time of the E2 after the start of the first E2	Plant	10.83	**0.0015**	0.1247	2.45	0.1217	0.0312	1.87	0.1753	0.0240
	Aphid	1.12	0.2933	0.0145	6.06	**0.0161**	0.0739	1.71	0.1956	0.0219
Relative amount of sE2 on E2	Plant	40.36	**0.0000**	0.3469	45.79	**0.0000**	0.3760	2.88	0.0940	0.0365
	Aphid	0.42	0.5185	0.0055	53.30	**0.0000**	0.4122	23.87	**0.0000**	0.2390
Percentage of probing spent in E2	Plant	19.18	**0.0000**	0.2015	0.89	0.3475	0.0116	0.36	0.5492	0.0047
	Aphid	20.93	**0.0000**	0.2160	0.06	0.8117	0.0008	0.11	0.7428	0.0014

^1^ Plant means the comparison of infected plants and uninfected plants; Aphid means the comparison of aphids with ascospores and aphids without ascospores. Bold *p*-values indicate significant main effects of cultivar, *Sclerotinia sclerotiorum* infection, or the interaction of the two on the EPG variables.

## Data Availability

The associated data produced for this study can be cited: [125].

## Supplementary Materials

The following supporting information can be downloaded at https://www.mdpi.com/article/10.3390/jof10030202/s1, Table S1: The definitions of the waveforms scored in the electropenetrography (EPG) analyses. Table S2: The main variables with significant difference between two oilseed rape cultivars.
