# Supplementary material for: Aphids May Facilitate the Spread of Sclerotinia Stem Rot in Oilseed Rape by Carrying and Depositing Ascospores
Source: J Fungi (Basel). 2024 Mar 6;10(3):202. doi: 10.3390/jof10030202 (PMC10971745; doi:10.3390/jof10030202)
Supplement: Supplementary file 1 [file jof-10-00202-s001.zip › jof-2869378-supplementary.pdf]

Table S1. The definitions of the waveforms scored in the electropenetrography (EPG) analyses

Acronym	Variable type	Definition
General		
n_Pr	Frequency	Number of probes
s_Pr	Time	Total probing time
s_nE	Time	Total duration of the no phloematic phase
s_np	Time	Total time of the non-probing intervals
s_np.1E	Time	Duration of the nonprobe period before the 1st E
t_1E2rec	Time	Time from the start of EPG to the 1st E2
t_1Erec	Time	Time from the start of EPG to the 1st E
Surface-mesophyll (Leaf)		
t_1Pr	Time	Time to the first probe from the start of EPG
n_Pr.1E1	Frequency	Number of probes before the first E1 (first phloem contact)
n_bPr	Frequency	Number of short probes (C < 3 minutes)
n_Pr.1E1	Frequency	Number of probes before the 1st E1
t_1C.1pd	Time	Time from the beginning of the 1st probe to the first pd
n_pd	Frequency	Number of pd
s_pd	Time	Total duration of pd
t_1EinPr	Time	Time from the beginning of that probe to the 1st E
s_C	Time	Total C duration with pd
%probtimeinC	Index	% of probing spent in C
Phloem		
s_E	Time	Total duration of the E phases
n_E1	Frequency	Number of E1 periods
s_E1	Time	Total duration of E1
d_E1followedby1sE2	Time	Duration of the E1 followed by the first sustained E2 (> 10 min)
s_E1followedbysE2	Time	Total duration of E1 followed by sustained E2 (> 10 min)
%_E1/E12	Index	Relative amount of E1 on E12
s_E2	Time	Total duration of E2 periods
s_longestE2	Time	Duration of the longest E2
E2index	Index	phloemian index: % of the time of the E2 after the start of the 1st E2
%sE2/E2	Index	Relative amount of sE2 on E2
%probtimeinE1	Index	% of probing spent in E1
%probtimeinE2	Index	% of probing spent in E2

Table S2. The main variables with significant difference between two oilseed rape cultivars.

Variables[1]	Xinyou17			Zheping4		
	Control	TI	TII	Control	TI	TII
**Overview**						
**n_Pr_1E1**	7.85±0.62	——	——	10.55±2.06	8.05±0.92	16.65±2.36
**n_Pr**	17.45±2.37	——	29.80±2.92	15.15±1.76	29.50±3.03	34.65±1.63
**s_Pr**	26764.39±216.80	27495.89±200.32	24162.40±625.27	27082.39±154.76	22715.67±852.01	20095.60±1119.60
**s_np.1E**	1133.67±184.08	——	——	1130.70±198.37	——	2090.33±314.79
**s_np**	2160.47±166.80*	——	4281.80±672.80	1420.49±145.65	5245.71±842.76	7394.94±1184.99
**s_nE**	16601.06±1704.89	10426.87±1638.01	——	18560.36±1377.40	——	——
**t_1Erec**	8693.10±1114.13	3267.90±431.22	5626.64±786.82	9798.92±1251.03	3954.45±346.31	——
**t_1E2.rec**	8812.00±1105.33	3199.31±379.87	5746.92±807.14	11140.57±1533.85	——	——
**Surface**						
**t_1Pr**	354.59±53.85*	88.52±12.53	132.11±33.83	141.20±23.67	80.00±9.43	——
**Mesophyll**						
**n_pd**	83.65±6.02*	——	217.25±15.62	122.95±11.61	193.85±19.18	187.70±13.40
**s_pd**	450.37±59.89	——	925.58±63.01	624.60±59.50	——	935.97±61.11
**n_bPr**	5.40±0.90	——	12.90±1.92	5.35±0.91	19.50±2.51	21.85±2.11
**t_1C.1pd**	782.10±107.88*	13.68±2.92	99.11±23.50	224.54±37.77	96.09±10.12	50.84±9.47
**t_1EinPr**	2488.52±182.91	783.02±67.35	722.92±53.35	2515.39±412.30	1111.03±87.39	1521.23±161.15
**s_C**	12462.70±1423.51*	8526.99±1511.72	——	18638.07±976.35	11317.04±1167.65	11758.48±775.57
**%probtimeinC**	46.11±6.11*	24.98±3.84	——	70.23±4.82	——	56.73±4.65
**Phloem**						
**n_E1**	1.40±0.11*	3.70±0.50	7.40±0.56	2.90±0.44	6.40±0.85	——
**s_E1**	149.24±10.89*	104.83±12.37	227.14±16.75	301.16±46.53	——	138.93±11.55
**d_E1followedby1sE2**	70.17±3.32*	41.49±3.85	30.79±1.25	83.18±2.00	31.82±1.52	29.99±1.41
**s_E1followedbysE2**	127.30±6.46	72.57±6.76	93.68±9.13	128.86±13.92	81.08±10.06	78.87±6.66
**%_E1/E12**	1.62±0.35*	——	1.98±0.14	4.29±0.73	12.29±2.33	2.05±0.26
**%probtimeinE1**	0.57±0.04*	0.43±0.05	0.95±0.06	1.04±0.18	——	0.50±0.07
**s_E2**	12890.69±1741.22	——	——	9042.12±1093.06	7808.03±1946.51	——

**s_longestE2**	11712.43±1711.32*	——	5593.51±676.01	6129.23±1881.79	1945.35±519.09	——
**%sE2/E2**	98.11±0.98*	77.09±4.28	47.11±3.31	75.33±5.46	33.17±6.23	——
**E2index**		——	50.06±2.78	57.30±6.03	37.51±7.94	——
**%probtimeinE2**	52.84±6.13*	——	——	29.90±5.44	——	——
**s_E**	12986.67±1579.42	——	——	8909.09±1266.80	——	——

[1] The table displays means ± standard error (SE) values. The data were compared using student's *t*-test or Mann-Whitney *U*-test after square-root transformation for the number of occurrences, natural log transformation for the duration, and square-root arcsine transformation for the proportion. The level of significance was set at $p < 0.05$. '——' indicates no statistical difference between control treatments and treatment I (TI)/treatment II (TII) in one cultivar. The symbol '*' in the control column indicates a significant difference between the control treatments of the two cultivars. The differences in EPG variables were compared between control (feeding behaviour of aphids not carrying ascospores on uninfected plants) and treatment I (feeding behaviour of aphids not carrying ascospores on infected plants) and between control and treatment II (feeding behaviour of aphids with adherent ascospores on uninfected plants) on the two cultivars.
